# Protective Effect of Zinc Oxide and Its Association with Neutrophil Degranulation in Piglets Infected with Porcine Epidemic Diarrhea Virus

**DOI:** 10.1155/2021/3055810

**Published:** 2021-06-23

**Authors:** Qian Zhang, Tao Wu, Siyuan Li, Yuxuan Meng, Zihan Tan, Mengjun Wu, Dan Yi, Lei Wang, Di Zhao, Yongqing Hou

**Affiliations:** Hubei Key Laboratory of Animal Nutrition and Feed Science, Wuhan Polytechnic University, Wuhan 430023, China

## Abstract

Porcine epidemic diarrhea virus (PEDV) has reemerged throughout the world in the past ten years and caused huge economic losses to the swine industry. No drugs are available to prevent or treat PEDV infection in piglets. Zinc oxide (ZnO) has been shown to reduce diarrhea. However, little is known about its role in PEDV infection. In this study, twenty-four 7-day-old piglets were randomly divided into three treatment groups: control, PEDV, and ZnO+PEDV. Piglets in the ZnO+PEDV group were orally administered with 100 mg/kg·BW ZnO and then inoculated PEDV at a dose of 10^4.5^ TCID_50_ (50% tissue culture infectious dose) per pig. Growth performance, histologic lesions, viral load, indicators of intestinal damage, inflammation, and oxidative stress were recorded or detected to determine the effect of ZnO on PEDV infection. And the underlying mechanisms were revealed by microarray and proteomic analyses. Results showed that ZnO administration mitigated diarrhea and the reduction of average daily weight gain induced by PEDV infection. ZnO could inhibit PEDV replication in the small intestine and colon. Both villus height and crypt depth were affected by PEDV infection in the duodenum and jejunum, which could be rescued by ZnO administration. Moreover, the activity of catalase was decreased both in plasma and intestine after PEDV infection, while increased in the intestine by ZnO administration. PEDV infection also significantly increased the concentration of H_2_O_2_ in jejunal and ileum and decreased the activity of total superoxide dismutase and glutathione peroxidase in plasma, whereas ZnO administration obviously increased the activity of total superoxide dismutase and decreased the concentration of H_2_O_2_ in the ileum. The concentrations of IL-1*β*, IL-6, and IL-8 in the plasma were all decreased upon ZnO administration. A large number of differentially expressed genes and proteins were identified in the ileum among the three groups by microarray and proteomic analyses. Gene Ontology and Reactome pathway analyses indicated that neutrophil degranulation and nutrient metabolism were the main biological process and pathways in both PEDV infection and ZnO administration. Overall, ZnO administration could improve growth performance, intestinal redox status, morphology, and function and reduce diarrhea in PEDV-infected piglets; ZnO could exert antiviral and anti-inflammatory effects on PEDV-infected piglets probably through regulating neutrophil degranulation. Our findings have important implications in piglet and infant nutrition.

## 1. Introduction

Porcine epidemic diarrhea virus (PEDV), which belongs to coronavirus, is one of the main pathogens that cause diarrhea in neonatal piglets [1]. From the 1990s onwards, porcine epidemic diarrhea (PED) was sporadic or localized outbreaks in Europe and Asia. Since 2010, PED has reemerged in China and other Asian countries with the appearance of highly pathogenic strains [1]. Most importantly, PEDV has been spread to North and South America for the first time in 2013 [2]. PED has become a worldwide problem in the past few decades and caused huge economic losses to the global swine industry [3]. PEDV mainly infects and replicates in villous enterocytes of the small intestine. Although other tissues, such as the lung, spleen, liver, and kidney could show PEDV positive by qPCR, there is no evidence that PEDV could replicate outside the intestinal tract [4]. Generally, vaccines are used to protect piglets against PEDV infection, and no specific drugs or curatives are shown to prevent or treat PEDV infection. Since high mutation rates of coronavirus may lead to the generation of more virulent genotypes and failure of vaccination [5], there is a growing need to develop alternative anti-PEDV agents with a different mechanism of action.

ZnO is an amphoteric oxide with a higher solubility at acidic pH. When ZnO reaches the stomach of piglets, most insoluble ZnO is transformed into Zn ions, and the remaining ZnO plays a role in the intestine [6]. Zn is an essential trace element, involved in the synthesis of more than 300 enzymes, and is closely associated with gut development and immune function [7]. In addition to Zn ions, ZnO molecule itself could also exert a positive effect on antibiotic resistance in bacteria [8]. Because of its antibacterial, disinfecting, and anti-inflammation properties, ZnO plays an important role in a wide range of applications, ranging from chemicals, pharmaceuticals to agriculture, and is used in various types of nutraceuticals and dietary supplements [9]. ZnO is unique and hard to be replaced even though ZnO has the potential to pollute the environment. Numerous studies have been conducted to find optimum methods to modify and improve the performance of ZnO [10]. However, so far, the effect of ZnO on PEDV infection and its mechanism of action are still unclear.

The increasing use of “-omics” technologies over the past decade greatly boosts the development of science. They make it easier to reveal the interactions of the host, pathogen, and other compounds to understand the complexity of animal biology and physiology [11]. Therefore, in the present study, we explored the mechanisms responsible for the anti-PEDV and anti-inflammatory effect of ZnO in newborn piglets through microarray and proteomic analyses. Our findings are expected to shed new light on the pathogenic mechanism of intestinal viral infections and provide guidance for the application of ZnO in piglet nutrition and even infant nutrition.

## 2. Materials and Methods

### 2.1. Animals

This study was approved by the Animal Care and Use Committee of Wuhan Polytechnic University. Twenty-four 7-day-old crossbred (Duroc × Landrace × Large White) healthy piglets with similar bodyweights were purchased from a PEDV-negative farm. Piglets were housed individually in three pens with strict control of cross-infection, and the ambient temperature was maintained around 25°C. The living environment was followed animal welfare guidelines in the whole experimental period. The experimental basal diet (liquid milk replacer) (Table 1), which was formulated to meet the requirements of nutrients for suckling piglets, was purchased from Wuhan Anyou Feed Co., Ltd. (Wuhan, China).

### 2.2. Experiment Design

All piglets were randomly divided into three treatment groups: control, PEDV, and ZnO+PEDV. The entire trial period was 11 days. During days 4 to 10 of the trial, the piglets in the ZnO+PEDV group were orally administered with 100 mg/kg·BW ZnO (purity ≥ 99%; dissolved in the liquid milk replacer), and piglets in the other two groups received the same volume of the liquid milk replacer. On day 8 of the trial, piglets in the PEDV and the PEDV+PR groups were orally inoculated PEDV at a dose of 10^4.5^ TCID_50_ (50% tissue culture infectious dose) per pig, while the control group was inoculated with the same volume of sterile saline solution [12]. Piglets were observed daily throughout the experiment period to record health status and diarrhea occurrence. Feces were scored as described by Zhang et al. [13]. Basically, feces were classified into four levels: 0 = normal, 1 = pasty, 2 = semiliquid, and 3 = liquid. Score ≥ 2 was considered diarrhea. On day 11, all piglets were orally administered with 10% D­xylose (1 mL/kg BW). One hour later, piglets were weighed and blood samples were collected from the anterior vena cava. Then, all piglets were sacrificed, and the intestinal samples were collected to be fixed with 4% paraformaldehyde or rapidly frozen in liquid nitrogen and stored at -80°C until analysis. The morphological structures of the intestine were observed as described previously [14].

### 2.3. Determination of D-Xylose, Diamine Oxidase (DAO), and Intestinal Fatty Acid-Binding Protein (I-FABP) in Plasma

The concentration of D-xylose and the activity of DAO in plasma were detected by colorimetric method using the kits from Nanjing Jiancheng Bioengineering Institute (Nanjing, China). The concentrations of I-FABP in the plasma were measured by using a commercial ELISA kit (R&D Systems Inc., CA, USA). All assays were performed according to the manufacturer's instructions.

### 2.4. Antioxidant Enzymes and Related Products in Plasma and Intestine

The activities of glutathione peroxidase (GSH-Px), catalase (CAT), total superoxide dismutase (T-SOD), and myeloperoxidase (MPO), as well as the concentrations of hydrogen peroxide (H_2_O_2_), were determined by using commercially available kits (Nanjing Jiancheng Bioengineering Institute, Nanjing, China) according to the manufacturer's protocols. Assays were performed in triplicate.

### 2.5. Enzyme-Linked Immunosorbent Assay (ELISA)

The concentrations of cytokines (IL-1*β*, IL-6, and IL-8) in the plasma of piglets were measured by using ELISA kits (RD Systems, Quantikine, USA) specific for swine. All assay procedures were performed according to the manufacturer's instructions.

### 2.6. Microarray Analysis

Microarray analysis was performed as described previously [13]. Briefly, a GeneAtlas® system (Affymetrix, Santa Clara, CA, USA) was employed. RNA extraction and quality control were performed as described previously [13]. Labeled fragmented single-stranded cDNA (ss-cDNA) was synthesized by using purified total RNA (100-500 ng) as the template following Affymetrix WT PLUS Labeling Assay protocol. Then, the mixtures of biotinylate labeled ss-cDNAs were hybridized onto porcine Gene 1.1 ST Arrays (Affymetrix, Santa Clara, CA, USA). After being washed by a fluidic station, the arrays were scanned with an imaging station in a GeneAtlas® system (Affymetrix, Santa Clara, CA, USA), and the acquired array raw data were analyzed with the Affymetrix Command Console Software Version 1.4. Quantile normalization and subsequent data processing were performed by the Affymetrix Transcriptome Analysis Console (TAC) Software 4.0. The cutoffs for differentially expressed genes (DEGs) were set at fold change > 1.5 or <0.67 and FDR < 0.01. Gene Ontology (GO) (http://www.geneontology.org/) and Reactome pathway database (http://www.reactome.org) were used for further analysis.

### 2.7. Protein Extraction, Digestion, and Nano-LC-MS/MS Analysis

150 mg of ileal tissue was homogenized with 1 mL of T-PER™ Tissue Protein Extraction Reagent (Thermo Fisher Scientific, USA) containing PMSF Protease Inhibitor (Roche, USA). The protein in the supernatant was collected by centrifugation and quantified by the bicinchoninic acid (BCA) assay. Then, 400 *μ*g proteins was subjected to filter-aided sample preparation (FASP) trypsin digestion to generate peptides according to the procedure described by Wu et al. [15]. The final peptides were desalted using Pierce C18 Tips (Thermo Fisher Scientific, USA) and quantified by Pierce™ Quantitative Colorimetric Peptide Assay (Thermo Fisher Scientific, USA) according to the manufacturer's protocols.

The nano-LC-MS/MS analysis was performed using the Q Exactive mass spectrometer (Thermo Fisher Scientific, USA), which was coupled with the Easy-Nano Ultimate 3000 UPLC system (Dionex, Thermo Fisher Scientific, USA). Briefly, 1 *μ*g peptides was loaded onto the trap column (100 *μ*m × 20 mm; Acclaim PepMap 100; Thermo Fisher Scientific, USA) and separated on the C18 capillary column (75 *μ*m × 150 mm; Acclaim PepMap RSLC, Thermo Fisher Scientific, USA). Then, the separated peptides were analyzed in the Q Exactive Orbitrap mass spectrometer. The MS data were acquired using the data-dependent acquisition (DDA) mode. In order to improve the effective utilization of the mass spectrometer, the parameters were set as follows: automatic gain control (AGC), 1 × 105 ions; signal threshold, 8000 ions/s; maximum injection time, 50 ms; dynamic exclusion duration, 40 s.

### 2.8. Identification of Differentially Expressed Proteins (DEPs) and Data Analysis

The raw MS data were further processed with the MaxQuant software (version 1.6.1.0, Max Planck Institute of Biochemistry in Martinsried, Germany) and mapped to the UniProtKB *Sus scrofa* database (22,191 sequences, downloaded November 10, 2018). The parameters were set as described previously [14]. The MaxQuant output files were analyzed by using the Perseus software (v.1.6.2.3, http://www.perseus-framework.org). The proteins with >1.5-fold change or <0.67- fold change and FDR < 0.05 were recognized as DEPs.

DEP lists were further processed with GO and Reactome databases for GO term and pathway enrichment analysis. The relationships among these DEPs were depicted in Venn diagrams (http://bioinformatics.psb.ugent.be/webtools/Venn/). Cluster analysis was performed by MetaboAnalyst 5.0 (https://www.metaboanalyst.ca/faces/home.xhtml).

### 2.9. Quantitative RT-PCR (qRT-PCR)

RNA was extracted by using TRIzol reagent (Takara, Dalian, China) according to the manufacturer's instructions. Extracted RNA (500 ng) was subsequently used for cDNA synthesized using PrimeScript®RT reagent Kit with gDNA Eraser kit (Takara, Dalian, China). Reactions of qPCR were set up using the SYBR®Premix Ex Taq™ (Takara, Dalian, China) and analyzed on the Applied Biosystems 7500 Fast Real-Time PCR System (Foster City, CA, USA). The fold change in gene expression was determined using the 2^-*ΔΔ*Ct^ method relative to the values in mock samples after normalization to housekeeping genes RPL4 and GADPH. The primers used in the present study were listed in Table 2.

## 3. Results

### 3.1. Effects of ZnO Administration on Growth Performance and PEDV Replication

**Figure 1:**
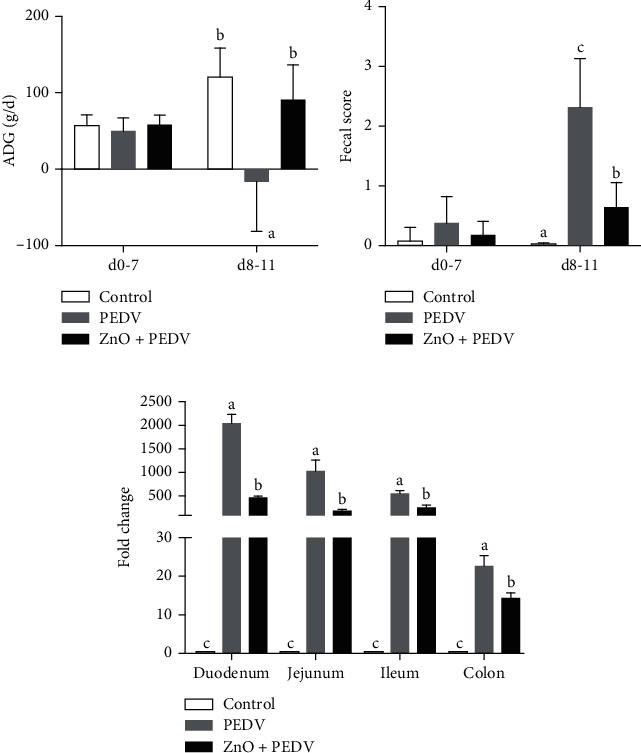
Effects of ZnO administration on the (a) average daily gain, (b) fecal score, and (c) PEDV replication in piglets infected with PEDV.

Prior to PEDV infection, there was no obvious difference in average daily weight gain (ADG), and no diarrhea was observed among the three groups (Figure 1(a)). PEDV infection significantly reduced (*P* < 0.001) the ADG of piglets, while the ADG of piglets in the ZnO+PEDV group were comparable to the piglets in the control group. Piglets in the PEDV group developed diarrhea after infection, which was indicated by the fecal score (>2), and ZnO administration significantly decreased the fecal scores compared with the PEDV group (*P* < 0.001) (Figure 1(b)).

To examine the effect of ZnO administration on PEDV infection, gene expression of the N gene of PEDV was detected in the small intestine and colon. Results showed that the N gene of PEDV was highly expressed in both PEDV and ZnO+PEDV groups, whereas no PEDV genes were detected in the control group (Figure 1(c)). The viral load was highest in the duodenum and lowest in the colon. However, the mRNA levels of N gene were obviously decreased in all these tissues of the ZnO+PEDV group when compared with the PEDV group.

### 3.2. Effects of ZnO Administration on Intestinal Morphology and Function

**Figure 2:**
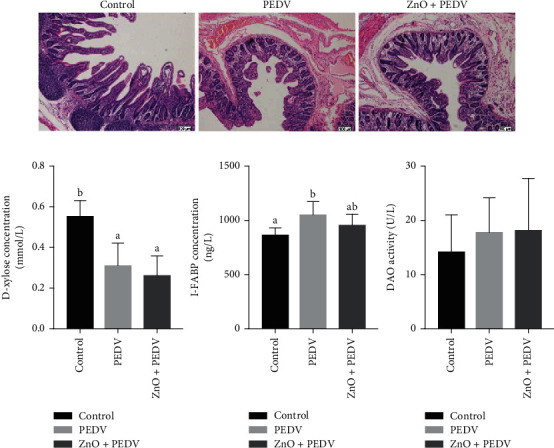
(a) Effects of ZnO administration on histopathological structure of the ileum (hematoxylin and eosin staining, ×100); (b) DAO activity and concentrations of D-xylose and I-FABP in plasma.

Histologic lesions were mainly observed in the ileum of PEDV-infected piglets, in which the intestinal villi were severely atrophied or shedding, and the administration of ZnO could significantly alleviate these lesions (Figure 2(a)). Meanwhile, villus height (VH), villus surface area, crypt depth (CD), and the ratio of VH/CD were measured (Table 3). Results showed that compared to the control group, the CD was increased and the VH/CD ratio was decreased (*P* < 0.05) in the duodenum and jejunum of the PEDV group. VH and villus surface area in the jejunum were also decreased in the PEDV group. ZnO administration could decrease CD and increase the VH/CD ratio in the duodenum and jejunum (*P* < 0.05), thereby improving intestinal function.

DAO activity and concentrations of D-xylose and I-FABP in plasma are important indicators for intestinal damage. In this study, PEDV infection significantly decreased D-xylose concentration (*P* < 0.001), but increased I-FABP concentration in plasma when compared with the control group (*P* = 0.005) (Figure 2(b)). ZnO administration to PEDV-infected piglets had a tendency to reduce the concentration of I-FABP, while there were no significant differences in the concentration of D-xylose and the activity of DAO between PEDV and ZnO+PEDV groups.

### 3.3. Antioxidative Effect of ZnO Administration on PEDV Infection

**Figure 3:**
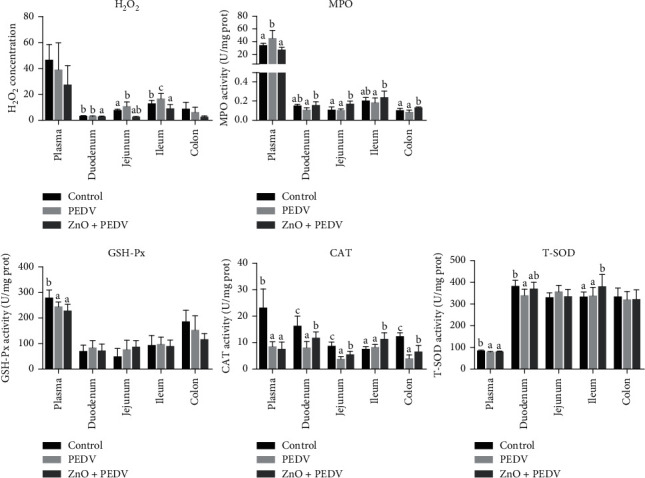
Antioxidative effect of ZnO administration in PEDV-infected piglets.

Compared with the control group, PEDV infection decreased the activity of GSH-Px in plasma (*P* < 0.05), the activity of CAT in plasma, duodenum, jejunum, and colon (*P* < 0.05), as well as the activity of T-SOD in plasma and duodenum (*P* < 0.05) (Figure 3). PEDV infection increased the concentration of H_2_O_2_ in jejunum and ileum. However, the ZnO+PEDV group increased the activity of CAT and MPO in the small intestine and colon, as well as the activity of T-SOD in the ileum when compared with the PEDV group. On the contrary, the activity of MPO in plasma and the content of H_2_O_2_ in the duodenum and ileum were obviously declined (Figure 3).

### 3.4. Anti-Inflammatory Effect of ZnO Administration on PEDV Infection

**Figure 4:**
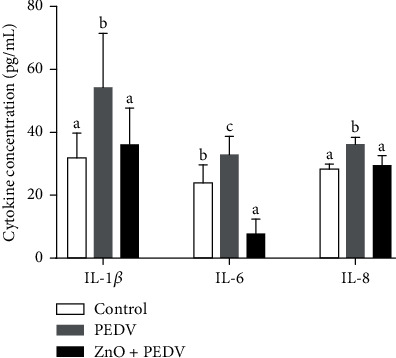
Anti-inflammatory effect of ZnO administration on PEDV infection.

The concentrations of IL-1*β*, IL-6, and IL-8 in the plasma were all increased in the PEDV group compared with the control group, while decreased in the ZnO+PEDV group compared with the PEDV group (Figure 4).

### 3.5. Identification and Analysis of DEGs in the Ileum

Compared to the control group, a total of 517 genes were upregulated and 379 downregulated in the PEDV group with cutoffs of fold change > 1.5 or <0.67 and FDR < 0.01. Only 2 DEGs (MT1A and MT-2B) were upregulated, and no DEGs were downregulated when the ZnO+PEDV group was compared with the PEDV group.

**Figure 5:**
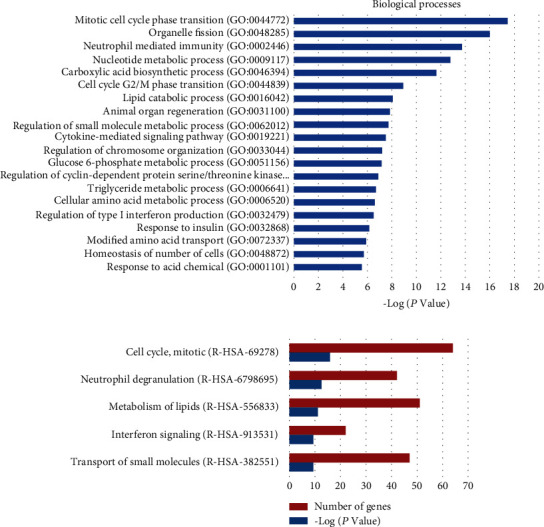
Analysis of the differentially expressed genes (DEGs) between the control and PEDV groups using the GO and Reactome databases. (a) Biological processes identified by GO enrichment. (b) The top 5 enriched Reactome pathways.

To explore whether the DEGs between control and PEDV groups share specific functional features, we performed enrichment analysis by using the GO database. The top significantly enriched biological processes were as follows: mitotic cell cycle phase transition, neutrophil-mediated immunity, nucleotide metabolic process, carboxylic acid biosynthetic process, lipid catabolic process, and regulation of small molecule metabolic process (Figure 5(a)). Reactome pathway analysis showed that these DEGs could be involved in cell cycle, neutrophil degranulation, lipid metabolism, and interferon signaling (Figure 5(b)).

### 3.6. Identification and Analysis of DEPs in the Ileum

**Figure 6:**
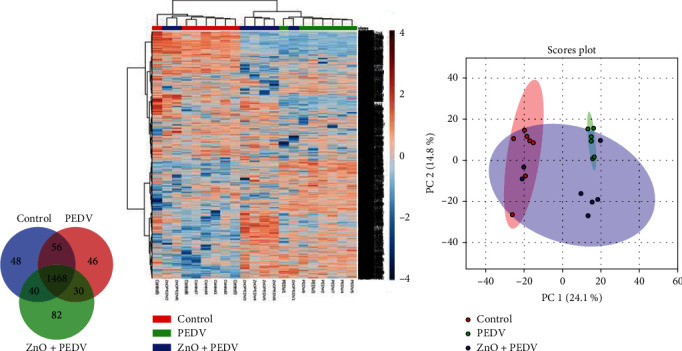
Overview of all differentially expressed proteins (DEPs) among control, PEDV, and ZnO+PEDV groups. The (a) Venn diagram showed the total number of identified proteins in these three groups. (b) Heat map and (c) PCA showed the convergence of the DEPs among different groups.

In this study, 1770 proteins were identified by LC-MS/MS platform, with 1612 proteins found in the control group, 1600 in the PEDV group, and 1620 in the ZnO+PEDV group. The analysis by Venn diagram showed that 1468 proteins were identified in all three groups (Figure 6(a)). Next, the heat map (Figure 6(b)) and principal component analysis (PCA) (Figure 6(c)) were performed to determine the convergence of the proteomics data. The results showed that proteins in the control and PEDV groups could be clearly distinguished, while the proteins in the ZnO+PEDV group could be distinguished from the control group and the PEDV group except for individual overlap.

**Figure 7:**
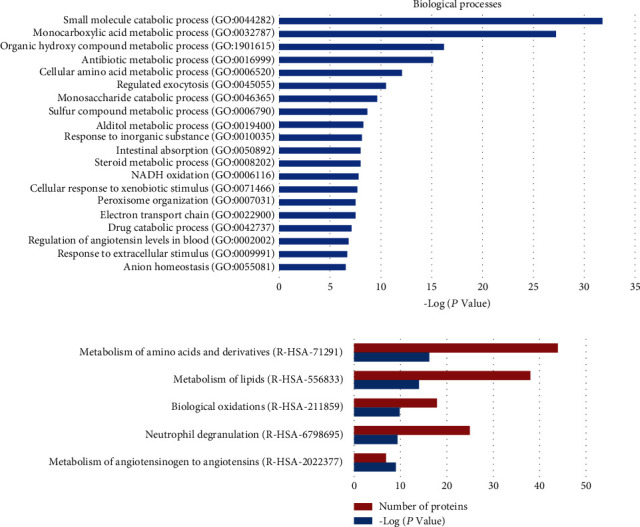
Analysis of the differentially expressed proteins (DEPs) between the control and PEDV groups using GO and Reactome databases. (a) Biological processes identified by GO enrichment. (b) The top 5 enriched Reactome pathways.

With the criteria of fold change > 1.5 or <0.67 and FDR < 0.05 (−log*Q* > 1.30), 284 DEPs were identified between the control and PEDV groups. Among them, 109 proteins were upregulated and 175 were downregulated in the ileum of the PEDV group. Then, these DEPs were subjected to biological process analysis, and the results showed that they were mainly enriched in metabolism-related processes (Figure 7(a)). Pathway analysis revealed that neutrophil degranulation and interferon signaling were significantly enriched apart from metabolism pathways (Figure 7(b)).

**Figure 8:**
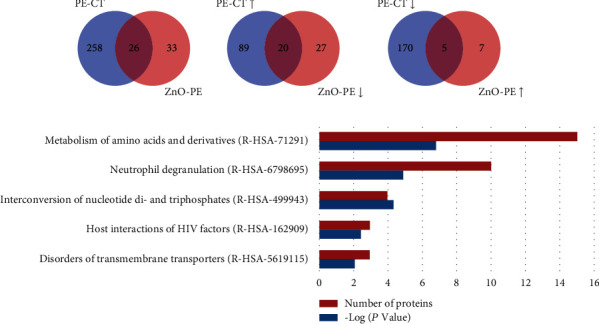
Comparison and analysis of DEPs identified in PEDV vs. control (PE-CT) and PEDV vs. ZnO+PEDV (ZnO-PE) groups. (a) The total number of DEPs in the PE-CT and ZnO-PE groups. The overlap indicated the number of common DEPs identified both in PE-CT and ZnO-PE groups. (b) The total numbers of upregulated DEPs in the PE-CT and downregulated DEPs in the ZnO-PE groups. The overlap indicated the numbers of commonly expressed proteins that upregulated in PE-CT and downregulated in ZnO-PE. (c) The total numbers of downregulated DEPs in the PE-CT and upregulated DEPs in the ZnO-PE groups. The overlap indicated the numbers of commonly expressed proteins that downregulated in PE-CT and upregulated in the ZnO-PE. (d) The top 5 enriched Reactome pathways among the DEPs of PEDV vs. ZnO+PEDV. ↑, upregulated; ↓, downregulated.

A total of 59 DEPs were identified between the PEDV group and ZnO+PEDV group with the same criterion above. Among them, 26 proteins were identified in the comparison between the control and PEDV groups (Figure 8(a)). After ZnO administration, 25 of the 26 proteins were restored, including 20 downregulated proteins and 5 upregulated proteins (Figures 8(b) and 8(c)). The Reactome pathway analysis revealed that DEPs between the PEDV group and ZnO+PEDV group were mainly involved in neutrophil degranulation and metabolism of amino acids and derivatives (Figure 8(d)).

### 3.7. Conjoint Analysis Based on Microarray and Proteomic Data

**Figure 9:**
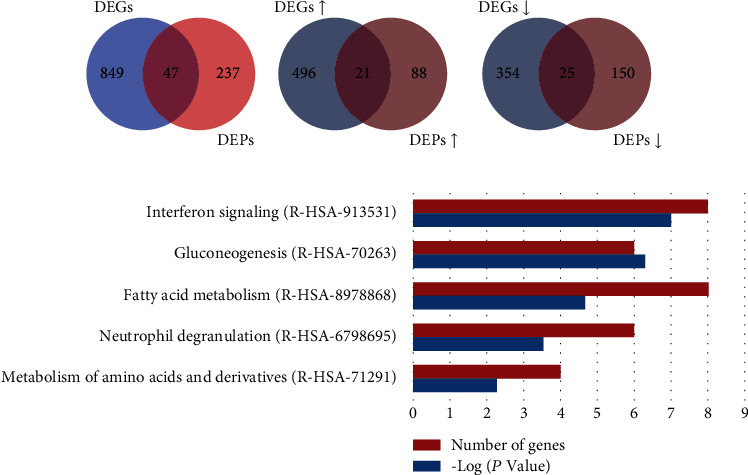
Comparison and analysis of DEGs and DEPs identified between the control and PEDV groups. (a) The total numbers of DEGs and DEPs identified between the control and PEDV groups. The overlap indicated the numbers of genes identified both in DEGs and DEPs. (b) The total numbers of upregulated DEGs and DEPs. The overlap indicated the number of upregulated genes identified both in DEGs and DEPs. (c) The total numbers of downregulated DEGs and DEPs. The overlap indicated the number of downregulated genes identified both in DEGs and DEPs. (d) The top 5 enriched Reactome pathways among genes identified both in DEGs and DEPs with the same expression pattern. ↑, upregulated; ↓, downregulated.

DEGs and DEPs between the control and PEDV groups were further analyzed. 47 DEPs were found to be significantly changed at the transcriptional level as well (Figure 9(a)). Among them, 46 genes had the same expression pattern in transcriptional and protein level, with 21 upregulated and 25 downregulated (Figures 9(b) and 9(c)). The Reactome pathway enrichment was well featured in interferon signaling, nutrient metabolism, and neutrophil degranulation (Figure 9(d)).

### 3.8. Validation of Neutrophil Degranulation-Associated Genes

**Figure 10:**
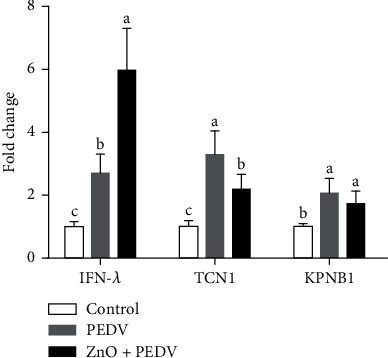
The measurement of neutrophil degranulation-associated genes by qPCR.

To validate the results of the microarray and proteomic data, we detected the expression of three neutrophil degranulation-associated genes (KPNB1, TCN1, and IFN-*λ*) by qPCR. As shown in Figure 10, all three genes were upregulated by PEDV infection. Compared with the PEDV group, the expression of IFN-*λ* in the ZnO+PEDV group further increased, while KPNB1and TCN1 decreased.

## 4. Discussion

PEDV is the main pathogen of lethal watery diarrhea in piglets, and the emergency of highly virulent strains makes classical vaccines unable to provide effective protection against PEDV [6]. Although a variety of compounds have been studied, the prevention and control of PED are still quite challenging [16, 17]. ZnO is a common nutritional enhancer with antibacterial, antidiarrhea and growth-promoting properties [9]. However, the effects of ZnO on diarrhea caused by PEDV are unclear. Therefore, we conducted this study to determine the role of ZnO and further elucidated the underlying mechanism using microarray and proteomic analyses.

In this study, PEDV infection dramatically decreased the ADG of piglets. This may be closely related to the intestinal dysfunction induced by PEDV, which was supported by the increase of diarrhea incidence. Similar findings were also found by Curry et al. and Wu et al. [15, 18]. ZnO administration substantially alleviated the ADG reduction caused by PEDV infection. This is consistent with the finding that ZnO could improve the growth performance. The beneficial effect of ZnO on growth performance is probably due to the fact that ZnO could promote food intake by stimulating ghrelin secretion from the stomach or improve intestinal absorption by altering the intestinal morphological structure [10, 19]. Consistent with the latter notion, ZnO administration alleviated diarrhea, as indicated by the decrease of fecal score in the present study.

The integrity of the intestinal epithelium is of great significance to maintaining intestinal function [20]. When the intestine is impaired, the concentration of D-xylose in plasma will decrease, while the concentration of I-FABP and the activity of DAO would increase [12]. In the present study, compared with the control group, PEDV infection decreased the concentration of D-xylose in plasma, but increased the concentration of plasma I-FABP, indicating that PEDV infection caused intestinal dysfunction. Since ZnO administration could slightly reduce the concentration of I-FABP in plasma, this indicated that ZnO has the potential to alleviate intestinal injury. Likewise, villus height and crypt depth are closely related to intestinal absorption and barrier function and would be important indicators of intestinal mucosal development [21]. Villus height and crypt depth were affected by PEDV infection in the duodenum and jejunum, which could be rescued by ZnO administration. Similarly, the beneficial effects of ZnO on intestinal function were also observed in weaning piglets [6, 10]. These results substantiated that ZnO could alleviate intestinal injury and improve intestinal function in PEDV-infected piglets.

H_2_O_2_ and MPO are important indicators of oxidative stress [22]. The concentration of H_2_O_2_ could reflect the degree of free radical accumulation, and high concentration of H_2_O_2_ will directly cause oxidative damage. SOD is an important antioxidant enzyme that facilitates the breakdown of the toxic superoxide radical into ordinary molecular oxygen (O_2_) or H_2_O_2_. H_2_O_2_ could be further degraded into H_2_O and O_2_ by CAT and GSH-Px [23]. In the present study, the activity of CAT was strongly affected. After infection, the activity of CAT in plasma and most intestinal segments decreased, while the intervention of ZnO increased the activity of CAT in the intestine. In addition, PEDV infection significantly increased the concentration of H_2_O_2_ in the jejunum and ileum and reduced the activity of SOD and GSH-Px in plasma. This is in concordance with our previous findings [12]. However, ZnO administration obviously increased the activity of SOD and decreased the concentration of H_2_O_2_ in the ileum. These results indicated that ZnO could effectively improve the redox status and alleviate oxidative damage in piglets infected with PEDV.

It is well known that intestinal immunity is regulated by a variety of cytokines, and inflammation could aggravate the pathogenesis of coronavirus. In line with our previous study [15], piglets infected with PEDV showed obvious inflammation, which could be reduced by the administration of ZnO. In fact, the effect of ZnO on inflammation is controversial. Several studies have revealed the anti-inflammatory effects of ZnO on pigs [6, 24]. On the other hand, proinflammatory effects were observed in piglets fed ZnO nanoparticles, which may be due to the production of ROS [25]. Song et al. reported that the expression of IL-6 and TNF-*α* did not differ between lipid-coated ZnO and basal ZnO groups [10]. These results indicate that the effect of ZnO on inflammation may be affected by its modification.

Although it is well known that ZnO has antibacterial properties, the effect of ZnO on viral replication is unclear. Surprisingly, ZnO intervention significantly reduced the mRNA level of the PEDV N gene. Importantly, ZnO administration decreased the expression of the N gene in the small intestine and colon, indicating that ZnO plays an antiviral effect in most of the intestine. Further studies are needed to reveal the relevant mechanism. Chai et al. investigated the effect of ZnO on TGEV infection and found that ZnO could exert an enhanced protective effect on the intestine and stimulate the systemic humoral immune response. Unfortunately, the authors did not detect TGEV replication after ZnO administration [26]. In this study, the expression of the PEDV N gene in the entire small intestine was extremely high, and the expression level in the duodenum was the highest. This is inconsistent with Jung et al.'s findings, which indicated that jejunal and ileal villus enterocytes were the main targets of PEDV replication [2]. The discrepancy may be due to the difference in detection method and strain. Meanwhile, we all found that the colon is also a target of PEDV.

Another important finding is that the neutrophil degranulation pathway was enriched with the help of microarray and proteomic analyses. Neutrophils could release granule-derived mediators, such as antimicrobial proteins, enzymes, and even reactive oxygen species through degranulation. Excessive degranulation of neutrophils is a common feature of many inflammatory disorders [27]. Conjoint analysis based on microarray and proteomic data showed that the genes related to neutrophil degranulation (such as KPNB1, CD44, MME, and VAT1) could be regulated at the transcriptional and protein levels. ZnO could also play a beneficial effect by restoring the expression of genes related to neutrophil degranulation (such as KPNB1 and TCN1). IFN-*λ*, which is mainly expressed in intestinal epithelial cells, is a unique immunomodulator that could reduce the degranulation of neutrophils [28]. ZnO administration significantly increased the expression of IFN-*λ*, suggesting that ZnO could exert an anti-inflammatory effect through the IFN-*λ*-mediated reduction of neutrophil degranulation during PEDV infection. Besides, IFN-*λ* elicited by ZnO may act on neutrophils to decrease oxidative stress and intestinal damage [28]. IL-8, another cytokine whose expression level was reduced by ZnO administration, also acts as a strong neutrophil chemoattractant [27]. Therefore, the role of ZnO in PEDV infection may be closely associated with neutrophil degranulation. MPO is an important biomarker for oxidative stress, which is mainly expressed in neutrophils [29]. It is a multifunctional protein with proinflammatory and anti-inflammatory effects. PEDV infection could increase the activity of plasma MPO, while ZnO administration could reduce the activity of plasma MPO, but increase the activity of intestinal MPO. It remains unknown why there is a divergence in the effect of ZnO administration on MPO. Further studies are warranted to address the role of MPO in ZnO intervention in PEDV infection. In addition, interferon signaling and nutrient metabolism pathways were enriched after PEDV infection. This is consistent with previous studies, which showed that PEDV infection affects the innate immune response and nutrient transport in the small intestine of piglets [13, 15].

## 5. Conclusions

ZnO administration could improve growth performance, intestinal redox status, morphology, and function and reduce diarrhea in PEDV-infected piglets. Microarray and proteomic analyses revealed that ZnO exerted antiviral, antioxidant, and anti-inflammatory effects on PEDV-infected piglets probably through regulating neutrophil degranulation. These findings decipher the pathogenic mechanism of PEDV infection and provide guidance for the application of ZnO in piglet nutrition and PED control.

## Figures and Tables

**Table 1:** Nutrient components of the milk replacer (as fed basis), 100%.

Items	Crude protein	Crude ash	Crude fiber	Moisture	Lysine	NaCl	Calcium	Total phosphorus
Milk replacer	≥20.0	≤9.0	≤1.0	≤10.0	≥1.4	0.3-1.5	0.4-1.1	≥0.3

**Table 2:** Primers used in this study.

Gene name	Sequences (5′-3′)
RPL4	GGAAACCGTCGCGAGA
	GCCCCAGAGACAGTT

GAPDH	CGTCCCTGAGACACGATGGT
	CCCGATGCGGCCAAAT

KPBN1	TGGCCCTACAAGGGATAGAA
	CAGTGCTCCCTTGGCATAAA

TCN1	CGTATAGCACAGGAGAAGCTATG
	CAGCGATTGGTAGACGGAATAC

IFN-*λ*	ACATCCACGTCGAACTTCAG
	CAGCCTTGGGACTCTTTCTT

PEDV-N	CGCAAAGACTGAACCCACTAACTT
	TTGCCTCTGTTGTTACTCGGGGAT

**Table 3:** Effects of ZnO administration on intestinal morphology in PEDV-infected piglets.

Items	Control	PEDV	ZnO+PEDV	*P* value
*Villus height (μm)*				
Duodenum	265 ± 55	227 ± 18	265 ± 41	0.126
Jejunum	276 ± 34^b^	153 ± 16^a^	137 ± 20^a^	<0.001
*Crypt depth (μm)*				
Duodenum	69 ± 8^a^	83 ± 12^b^	70 ± 7^a^	0.012
Jejunum	67 ± 11^a^	104 ± 22^b^	56 ± 11^a^	<0.001
*Villus height/crypt depth*				
Duodenum	3.76 ± 0.59^b^	2.79 ± 0.66^a^	3.90 ± 0.35^b^	0.001
Jejunum	4.15 ± 0.74^c^	1.63 ± 0.32^a^	2.59 ± 0.26^b^	<0.001
*Villous surface area (μm^2^)*				
Duodenum	6966 ± 1850	5913 ± 537	6383 ± 1305	0.312
Jejunum	6785 ± 1325^b^	3484 ± 463^a^	4215 ± 1538^a^	<0.001

Values are mean and pooled SEM, *n* = 8. ^a,b,c^Within a row means with different superscripts differ, *P* < 0.05.

## Data Availability

The data used to support the findings of this study are available from the corresponding author upon request.
